# Predictive validity of admission criteria in predicting academic performance of medical students: A retrospective cohort study

**DOI:** 10.3389/fmed.2022.971926

**Published:** 2022-09-08

**Authors:** Amer Almarabheh, Mohamed Hany Shehata, Abdulrahman Ismaeel, Hani Atwa, Ahmed Jaradat

**Affiliations:** ^1^Department of Family and Community Medicine, College of Medicine and Medical Sciences, Arabian Gulf University, Manama, Bahrain; ^2^Department of Family Medicine, Faculty of Medicine, Helwan University, Helwan, Egypt; ^3^Department of Microbiology, College of Medicine and Medical Sciences, Arabian Gulf University, Manama, Bahrain; ^4^Department of Medical Education, College of Medicine and Medical Sciences, Arabian Gulf University, Manama, Bahrain; ^5^Department of Medical Education, Faculty of Medicine, Suez Canal University, Ismailia, Egypt

**Keywords:** predictive validity, admission criteria, academic performance, foundation year, medical program

## Abstract

**Introduction:**

Admission to medical school is one of the most competitive entry points in higher education. Medical school admissions committees need accurate and precise screening tools to select among well-qualified applicants. This study explores data from a cohort of graduated medical students over 6 years to offer a critical perspective on predictive validity in medical school admissions.

**Methods:**

A retrospective cohort study of 160 students was performed to identify the predictive validity of admission criteria for medical students to predict academic performance over 6 years for a cohort of all students enrolled in the medical program during the academic year 2013–2014.

**Results:**

The main results indicated that there was a statistically significant positive correlation between the admission criteria and Students’ performance in Year 1, Year 4, B.Sc. (Bachelor of Medical Science) exam, and Medical Doctor (MD) exam across the 6 years of the medical program, except for the English Test, which showed that there was no significant correlation with average MD exam scores for students who enrolled directly in Year 1. The results related to students who were admitted to the Foundation Program showed that there was no significant correlation between high school Grade Point Average (HSGPA) and their academic performance in Year 1, Year 4, B.Sc. exam, and MD exam. The overall results related to all study samples indicated that all predictor variables correlate significantly with all outcome variables (academic performance), and the results showed that Science test scores demonstrated 27.7, 15.0, 19.7, and 12.6% of variation in Students’ performance in Year 1, Year 4, B.Sc. exam, and MD exam, respectively.

**Conclusion:**

Science test scores were found to be more predictive of academic performance compared to other predictors. Not all the admission criteria used for student selection are good indicators of their achievement in the medical program. It is recommended that other valid and reliable admission tools, such as the multiple mini-interviews and the questionnaire for a candidate’s suitability to follow a problem-based learning curriculum, should be considered.

## Introduction

The number of qualified applicants for medical degree programs sometimes outstrips the number of available seats, making the selection process very competitive. Thus, the selection process must be clear and defendable to balance the supply and demand reasonably and consistently. In addition, it is logical that medical schools strive to select students who may be more inclined to become excellent physicians (1).

The criteria for selecting medical students are debatable, and selection techniques for admission from the substantially bigger applicant pool constitute a significant difficulty (2). Selection techniques are essential to finding individuals with the appropriate aptitude to finish the program and become safe physicians. In fact, such methods must rate students for admittance to the available number of seats. However, establishing the extent to which a specific tool anticipates its predictive validity or outcome measures is most challenging. There are many reasons for this, including deciding the most accurate outcome to validate the tool against; uncertainty on how candidates who achieve lower scores would have fared (3), and how to combine those tools to improve their predictive validity (1, 4).

When it comes to medical school admissions, there is no shortage of information. More students are applying for admission, and admission officers are becoming more stringent in their selection process because of the increasing quantity and quality of potential candidates and the limited number of available seats. A number of studies have questioned the predictive efficacy of different pre-admission indicators on student academic achievement. Consequently, more testing and modification of pre-admission factors are needed to eliminate improper selection criteria that are weakly predictive of the future performance of medical graduates (5, 6).

College performance indicators are often used in predictive validity studies to examine pre-admission characteristics (7). Grade point average (GPA) is a common college performance metric because of its perceived importance in measuring Students’ in-course college achievement. Although the GPA is widely accepted as a credible and accurate indicator of academic performance (8, 9), medical schools have yet to corroborate this. Grade inflation and institutional inequalities in grading are among the serious flaws in this kind of performance metric that may have a negative impact on its validity and reliability (9). Admission examinations for colleges and universities have been the subject of much discussion and debate in the academic literature (10). Pre-university assessments such as high school GPA, aptitude, and achievement tests were studied to see whether they predicted Students’ college GPA, and high school GPA was proven to be a better predictor of college GPA than preparatory examinations (11). On the contrary, Al-Rukban et al. found that the high school grade had less predictive power when it came to Students’ college GPAs (12). Murshid conducted another intriguing investigation to determine the effectiveness of admission criteria in predicting Students’ academic performance (13). He discovered that high school grades and achievement tests were both strong predictors of college GPA. Furthermore, in a recent study conducted by Alamoudi et al. (14) on the relationship between admission criteria and academic performance in basic science courses in health science colleges in a Saudi university, no significant correlation was found between admission exam scores and Students’ academic achievement. Similar studies performed in the US revealed that the Medical College Admission Test (MCAT) was a good predictor of the performance of medical graduates in the first 2 years of college (15, 16). The same trend was seen in the clinical years, where the students who did well in the MCAT performed better in the clinical years and in the residency program (17, 18). Admission procedures and aptitude tests in medical education have been studied extensively for their predictive value. For up to 23% of the variance in medical school performance, a systematic review of more than 150 papers on the relationship between admissions variables and success in medical schools was conducted and discovered that past academic performance was a modest predictor of college success (1).

The goal of this study was to verify if the admission criteria at Arabian Gulf University (AGU) were predictive of Students’ academic achievement during a 6-year program. This is the first study at AGU to look at this topic.

## Materials and methods

### Study context

The College of Medicine and Medical Sciences, Arabian Gulf University (CMMS-AGU) in Bahrain is a regional institution that provides medical education for students from Gulf Cooperation Council (GCC) countries, namely Bahrain, Saudi Arabia, Kuwait, Oman, United Arab Emirates, and Qatar. Currently, around 1,200 students are enrolled in all study years. The medical program is almost completely delivered in English. Therefore, students who are admitted to the college are required to achieve excellent scores in an English language test prior to acceptance to make sure that they meet the required language skills. Candidates who fail to demonstrate adequate English language proficiency are offered a 1-year foundation program where they receive intensive English language instruction.

The curriculum of the CMMS-AGU follows an integrated, problem-based approach. The medical program is offered in three phases. Phase I of the program extends over the first year of the program and mainly introduces basic science courses (medical physics, biology, biochemistry, and biostatistics) in addition to other general courses, including Islam, medical ethics, English language, computer science, social science, and psychology. Phase II of the program—or the pre-clerkship phase—focuses on basic medical sciences. This phase extends from year 2 to year 4 of the program and is fully integrated and problem-based. Phase III of the program is the clinical phase, where students receive clerkship clinical training in several training sites. Students are awarded the Doctor of Medicine (MD) degree following successfully completing the final MD exam at the end of Phase III.

### Study design, participants, and data collection

This is a retrospective cohort study conducted on 160 medical students who were enrolled in the CMMS-AGU in the academic year 2013–2014 and graduated in the academic year 2018–2019 to study the relationship between their performance during the study years and the admission criteria to determine the most predictive admission criteria that predict their performance (Table 1).

**TABLE 1:** Demographic data of the graduate students (*n* = 160).

Characteristic	*N* (%)
**Gender**	
Male	55 (34.4%)
Female	105 (65.6%)
**Admission status**	
Foundation program (Pre-year 1)	49 (30.6%)
Year 1	111 (69.4%)
**Nationality**	
Bahrain	66 (41.3%)
Kuwait	56 (35%)
Saudi	29 (18.1%)
Oman	9 (5.6%)
**Type of school**	
Government	126 (78.8%)
Private	34 (21.2%)
**Total**	**160 (100%)**

The collected data included Students’ demographics, pre-admission variables [high school grade point average (HSGPA), AGU-MCAT (biology, chemistry, physics, and mathematics) scores, and their English language test scores], and college academic performance indicators (Year 1 GPA, Year 4 GPA, average B.Sc. (Bachelor of Medical Science) exam scores, and average MD exam scores). AGU-MCAT (Science Test) and English language test are collectively called the “Entrance Exam.”

The Science Test covers basic topics in biology, chemistry, physics, and mathematics, which consists of a hundred A-type multiple-choice questions. Biology represents 40% of the exam questions, while chemistry, physics, and mathematics each represent 20%. Candidates are allowed a total of 3 h to complete the test.

The English language tests aim to assess the proficiency of students in English (reading, listening, and writing).

An overall total score is reported (100 points) for each test (Science test and English language test).

Multiple filters are used to screen candidates as follows: only students with an overall high-school score of at least 90% and an overall average score of at least pass in English Language Tests and Science Test are allowed to apply for year 1 or foundation year program. Candidates then undergo a semi-structured personal interview to evaluate their personality traits to determine the suitability of each candidate to study medicine and the extent to which they will be suited to the study program at the AGU, in which they are rated only as “pass” or “fail.” Candidates who pass the interview are then listed according to their balanced percentage (high school GPA and scores of Science tests and English language tests) in descending order, and those at the top of the list are admitted to the year 1 program, while others are admitted to the foundation year program depending on the available seats.

### Predictor variables

In addition to the high school GPA, predictor variables included the Science test and English language tests.

### Performance outcomes

These included the GPA of the final exams of year 1 (Year 1 GPA) and year 4 (Year 4 GPA), in addition to a comprehensive assessment of basic medical sciences after completion of the first 4 years of the program (average B.Sc. scores) and an exit assessment of all the clinical sciences studied during the clerkship phase (average MD scores).

### Statistical analysis

The data were entered and analyzed using SPSS (Chicago, IL, United States) software version 28. Qualitative variables were presented as frequencies and percentages, and quantitative variables were presented as means and SD. Pearson correlation coefficients were used to test the relationship between the admission criteria (predictor variables) and academic performance (outcome variables). Multiple linear regression analysis was performed for each outcome variable with all variables included. Stepwise multiple linear regression analysis was used to verify the predictive value of admission criteria. Standard coefficients with 95% confidence intervals, SE, and *R*^2^-values (coefficient of determination) were tabulated. In addition, standardized coefficients (β-values) were presented to show the relative independent contribution of the predictor variables. The regression model was tested using partial least squares structural equation modeling (PLS-SEM) and using Smart PLS version 3.2.7. A *p*-value of less than 0.05 was considered statistically significant.

### Ethical considerations

This research was approved by the Research and Ethics Committee of CMMS College of Medicine and Medical Sciences, AGU, Bahrain (Approval Number: E040-PI-2/21). The Research and Ethics Committee waived the need to obtain informed consent since this study used secondary data from the participants. All authors confirm that all methods were performed in accordance with the relevant guidelines and regulations.

## Results

### Demographic data

This cohort of students was enrolled at CMMS-AGU in the academic year 2013–2014. Out of 160 students, 105 (65.6%) were female students, and 55 (34.4%) were male students. Regarding the admission status, the majority of the students (69.4%) were accepted in Year 1 of medical school, while 49 students (30.6%) were accepted in the foundation program (Pre-Year 1). The enrolled students were Bahraini (41.3%), Kuwaiti (35%), Saudi (18.1%), and Omani (5.6%). The majority of the students (78.8%) came from governmental schools, while only 21.2% came from private schools (Table 1).

### Descriptive statistics and correlation matrix of study variables

The Students’ high school grades and their entrance exam scores (which are used as admission criteria) along with Students’ performance in Year 1, Year 4, B.Sc. exam, and MD exam are presented as means and SD. Results show that although the mean high school grades were high, the mean grades of the entrance exams were low (less than 60%) with a large variation (19.32) in the English test scores compared to the Science test scores (11.36). Students’ performance at the end of Year 1 (81.94 ± 6.89) and Year 4 (77.66 ± 6.58) was better than in B.Sc. (71.24 ± 7.38) and MD (71.99 ± 6.60) exams, as the latter two are comprehensive exams (Table 2).

**TABLE 2:** Means, standard deviations, and correlation matrix of study variables.

		Intercorrelations, Pearson correlations coefficients						
Variables	Mean (±SD)	HSGPA	Science test scores	English test scores	Year 1 GPA	Year 4 GPA	Average B.Sc. scores	Average MD scores
HSGPA	96.40 (±2.32)	1	0.199*	−0.017	0.288**	0.260**	0.257**	0.224**
Science test scores	57.19 (±11.36)		1	0.379**	0.527**	0.387**	0.444**	0.355**
English test scores	59.01 (±19.32)			1	0.463**	0.296**	0.318**	0.263**
Year 1 GPA	81.94 (±6.89)				1	0.718**	0.766**	0.616**
Year 4 GPA	77.66 (±6.58)					1	0.941**	0.841**
Average B.Sc. scores	71.24 (±7.38)						1	0.848**
Average MD scores	71.99 (±6.60)							1

*Correlation is significant at the 0.05 level (2-tailed).

**Correlation is significant at the 0.01 level (2-tailed).

The correlation study indicated that all predictor variables correlate significantly with academic performance (Year 1 GPA, Year 4 GPA, average B.Sc. exam scores, and average MD exam scores). This is an indication of the validity of these tests to predict the performance of medical students in medical school. Science test scores showed the strongest correlation with all academic performance indicators, while HSGPA showed the lowest correlation with all academic performance indicators.

The correlation between the English test and high school GPA (HSGPA) was a weak negative, statistically insignificant correlation (-0.017). Also, the results showed that the correlation between Year 4 GPA and average B.Sc. exam scores was the highest (*r* = 0.941) compared to the correlations of the predictors and outcome variables. The strongest correlation between two predictor variables was between Science test scores and English test scores (*r* = 0.379), and the shared variance between these two variables was about 14.4% (*R*^2^ = 0.1436).

### Relationship between predictor variables and academic performance

The correlation between admission exam scores and Students’ performance scores according to the admission type is presented in Table 3. For students who were ineligible to join Year 1 and were enrolled in an extra Pre-Year 1 foundation program, the results showed no statistically significant correlation between HSGPA and their academic performance in Year 1, Year 4, B.Sc. exam, and MD exam. In contrast, a moderate positive statistically significant correlation (ranging from *r* = 0.298 to *r* = 0.392) between HSGPA and academic performance in Year 1, Year 4, B.Sc. exam, and MD exam was noted for students who were enrolled directly in Year 1.

**TABLE 3:** Pearson correlation coefficients for predictor variables and academic performance (outcome indicators) according to admission types (foundation program and year 1 admission).

	Foundation program (*n* = 49)			Year 1 (*n* = 111)		
	HSGPA	Science test scores	English test scores	HSGPA	Science test scores	English test scores
Year 1 GPA	0.116	0.594**	0.576**	0.392**	0.433**	0.355**
Year 4 GPA	0.029	0.554**	0.449**	0.373**	0.281**	0.203*
Average B.Sc. scores	0.018	0.578**	0.465**	0.369**	0.350**	0.208*
Average MD scores	0.078	0.491**	0.371**	0.298**	0.252**	0.145

*Correlation is significant at the 0.05 level.

**Correlation is significant at the 0.01 level.

Positive, statistically significant correlations were found between English test scores and Science test scores on one hand, and Students’ performance in Year 1, Year 4, B.Sc. exam, and MD exam, on the other hand, for the students who went through the foundation program. This was found to be true also for those students who were directly enrolled in Year 1, except for a statistically insignificant positive correlation between English test scores and average MD exam scores. The correlation was consistently stronger for students who were enrolled in the foundation program than those for students who were directly enrolled in Year 1. Moreover, Science test scores have higher correlation coefficients than English test scores for both admission types.

### Regression analysis

A multiple regression model was fitted to study the linear relationship between Students’ performance in Year 1, Year 4, B.Sc. exam, and MD exam on the one hand and their admission criteria scores (HSGPA, English test scores, and Science test scores) on the other hand.

Regression analysis indicated that there is a statistically significant linear relationship between Students’ performance and admission scores. The amounts of variation in Students’ performance in Year 1, Year 4, B.Sc. exam, and MD exam explained by the predictors (adjusted *R*^2^) was 39.5, 20.3, 24.5, and 15.9%, respectively.

The values of the coefficients β fell from Year 1 to MD but were consistently higher for Year 1 GPA than Year 4 GPA, average B.Sc. exam scores, and average MD exam scores for each admission criteria. The values of the coefficients β for Science test scores were consistently higher than those for HSGPA and English test scores; this indicates that the Science test is one of the most important admission criteria in explaining variation in academic performance compared to the other predictors (Table 4).

**TABLE 4:** Regression modeling of the relationship between admission criteria and academic performance.

	Year 1 GPA	Year 4 GPA	Average B.Sc. scores	Average MD scores
HSGPA (coeff.)	0.662	0.594	0.617	0.501
(95% C.I.)	0.291–1.033	0.187–1.001	0.173–1.061	0.082–0.920
SE (β)	0.188 (0.223)	0.206 (0.209)	0.225 (0.194)	0.212 (0.176)
*p*-value	*p* < 0.01	*p* < 0.01	*p* < 0.01	*p* = 0.019
Science test scores (coeff.)	0.216	0.157	0.215	0.149
(95% C.I.)	0.134–0.298	0.067–0.247	0.117–0.313	0.056–0.241
SE (β)	0.041 (0.357)	0.045 (0.271)	0.050 (0.331)	0.047 (0.256)
*p*-value	*p* < 0.001	*p* < 0.01	*p* < 0.001	*p* < 0.01
English test scores (coeff.)	0.118	0.067	0.075	0.058
(95% C.I.)	0.071–0.166	0.015–0.119	0.019–0.132	0.004–0.111
SE (β)	0.024 (0.332)	0.026 (0.197)	0.029 (0.196)	0.027 (0.169)
*p*-value	*p* < 0.001	*p* = 0.011	*p* = 0.010	*p* = 0.034
Adjusted *R*^2^	39.5%	20.3%	24.5%	15.9%

#### Stepwise regression analysis

Stepwise multiple regression analysis was employed to identify and select the predictor variable that explains more variation in the academic performance indicator of medical students. Science test scores, English test scores, and HSGPA were entered into the regression model but in different steps (Table 5).

**TABLE 5:** Summary of stepwise regression analyses of predicting academic performance (*n* = 160).

Outcome variables	Model	Variables entered	Beta	*R*	*R* ^2^	Δ*R*^2^*	*F*	*P*-value
Year 1 GPA	1	Science test scores	0.527	0.527	0.277	0.277	60.652	*p* < 0.001
	2	Science test scores	0.410	0.599	0.359	0.082	43.911	*p* < 0.001
		English test scores	0.308					
	3	Science test scores	0.357	0.636	0.405	0.046	35.545	*p* < 0.001
		English test scores	0.332					
		HSGPA	0.223					
Year 4 GPA	1	Science test scores	0.387	0.387	0.150	0.150	27.835	*p* < 0.001
	2	Science test scores	0.349	0.430	0.185	0.035	17.773	*p* < 0.001
		HSGPA	0.190					
	3	Science test scores	0.271	0.464	0.215	0.030	14.463	*p* < 0.001
		HSGPA	0.209					
		English test scores	0.197					
Average B.Sc. scores	1	Science test scores	0.444	0.444	0.197	0.197	38.740	*p* < 0.001
	2	Science test scores	0.409	0.476	0.226	0.029	22.976	*p* < 0.001
		HSGPA	0.175					
	3	Science test scores	0.331	0.507	0.257	0.031	18.190	*p* < 0.001
		HSGPA	0.194					
		English test scores	0.196					
Average MD scores	1	Science test scores	0.355	0.355	0.126	0.126	22.764	*p* < 0.001
	2	Science test scores	0.323	0.388	0.151	0.025	13.912	*p* < 0.001
		HSGPA	0.160					
	3	Science test scores	0.256	0.416	0.173	0.022	11.007	*p* < 0.001
		HSGPA	0.176					
		English test scores	0.169					

*ΔR^2^: Change in R^2^.

##### Year 1 grade point average

Science Test scores were entered first as predictors into model 1, which accounted for significant variation and explained 27.7% of the variation in Year 1 GPA. Thus, Science test scores were more important in explaining the variation in Year 1 GPA than English Test scores and HSGPA [*R*^2^ = 0.277, *F*(1, 158) = 60.652, *p* < 0.001]. When English test scores were added as a second predictor in model 2, the two variables (Science test and English test) accounted for significant variation in Year 1 GPA [*R*^2^ = 0.359, *F*(1, 158) = 43.911, *p* < 0.001], and the prediction of Year 1 GPA has improved by 8.2% (change in *R*^2^ = 0.082). When HSGPA was added as a third predictor in model 3, the prediction of Year 1 GPA has improved by 4.6% [change in *R*^2^ = 0.046, *F*(1, 158) = 35.545, *p* < 0.001].

##### Year 4 grade point average

Science test scores were entered first as a predictor into model 1, which accounted for significant variation and explained 15.0% of the variation in Year 4 GPA. Thus, Science test scores were more important in explaining the variation in Year 4 GPA than English Test scores and HSGPA [*R*^2^ = 0.150, *F*(1, 158) = 27.835, *p* < 0.001]. When HSGPA was added as a second predictor in model 2, the two variables (Science test and HSGPA) accounted for significant variation in Year 4 GPA [*R*^2^ = 0.185, *F*(1, 158) = 17.773, *p* < 0.001], and the prediction of Year 4 GPA has improved by 3.5% (change in *R*^2^ = 0.035). When English test scores were added as a third predictor in model 3, the prediction of Year 4 GPA has improved by 3.0% [change in *R*^2^ = 0.030, *F*(1, 158) = 14.463, *p* < 0.001].

##### Average bachelor of medical science exam scores

Science test scores were entered first as a predictor into model 1, which accounted for significant variation and explained 19.7% of the variation in the average B.Sc. exam scores. Thus, Science test scores were more important in explaining the variation in the average B.Sc. exam scores than English test scores and HSGPA [*R*^2^ = 0.197, *F*(1, 158) = 38.740, *p* < 0.001]. When HSGPA was added as a second predictor in model 2, the two variables (Science test and HSGPA) accounted for significant variation in the average B.Sc. exam scores [*R*^2^ = 0.226, *F*(1, 158) = 22.976, *p* < 0.001], and the prediction of the average B.Sc. exam scores has improved by 2.9% (change in *R*^2^ = 0.029). When English test scores were added as a third predictor in model 3, the prediction of average B.Sc. exam scores has improved by 3.1% [change in *R*^2^ = 0.031, *F*(1, 158) = 18.190, *p* < 0.001].

##### Average medical doctor exam scores

Science Test scores were entered first as a predictor into model 1, which accounted for significant variation and explained 12.6% of the variation in the average MD exam scores. Thus, Science test scores were more important in explaining the variation in the average MD exam scores than English test and HSGPA [*R*^2^ = 0.126, *F*(1, 158) = 22.764, *p* < 0.001]. When HSGPA was added as a second predictor in model 2, the two variables (Science test and HSGPA) accounted for significant variation in average MD exam scores [*R*^2^ = 0.151, *F*(1, 158) = 13.912, *p* < 0.001], and the prediction of the average MD exam scores has improved by 2.5% (change in *R*^2^ = 0.025). When English test scores were added as a third predictor in model 3, the prediction of the average MD exam scores has improved by 2.2% [change in *R*^2^ = 0.022, *F*(1, 158) = 11.007, *p* < 0.001].

##### Combined admission criteria

**FIGURE 1:**
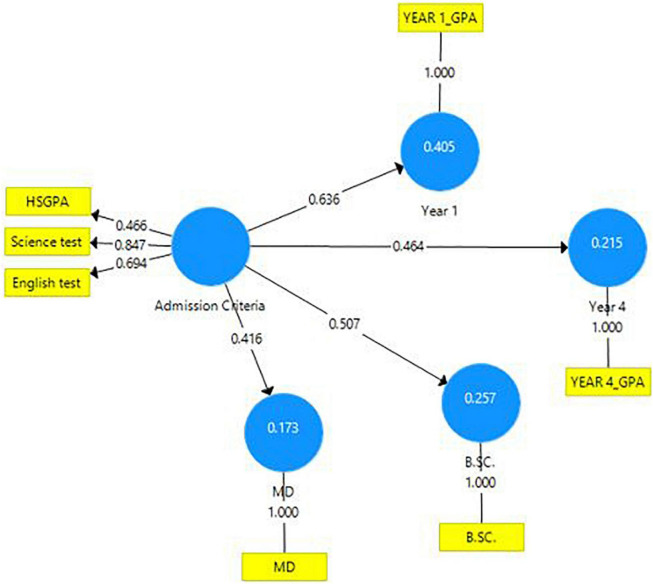
Regression model for predicting academic performance according to admission criteria.

From the stepwise multiple regression analysis (Table 5), the three admission criteria (HSGPA, English test scores, and Science test scores) together explained 40.5, 21.5, 25.7, and 17.3% of the variation in student performance in Year 1 GPA, Year 4 GPA, B.Sc. exam, and MD exam, respectively (Figure 1).

## Discussion

This study was conducted to verify the predictive validity of the admission criteria used in the CMMS-AGU. It explored the admission data (as predictor variables) and the performance of students in Year 1 final exam (represented by Year 1 GPA), Year 4 final exam (represented by Year 4 GPA), B.Sc. exam (a comprehensive assessment of basic medical sciences after completion of the first 4 years of the curriculum), and MD exam (an exit assessment of all the clinical sciences studied during the clerkship phase). Participating students in this study were both men and women who came from four Gulf countries.

This study found that there is a statistically significant correlation between most of the predictor variables and the performance indicators. The highest correlation was found to be with the scores of the Science test (part of the admission exam). Robi (19) found similar results, although they reported that HSGPA was superior to the entrance exam as a predictor of Students’ performance in medical school. The findings are partly consistent with a study by Mercer and Puddey (20), who found a highly statistically significant correlation between high school scores and the weighted average in the first 3 years of the medical program, and Alkushi and Althewini (21), who found that high school grades were the best predictor of college performance for women, while scholastic achievement admission test scores were a better predictor of college performance for men. This is also consistent with the findings of two studies (22, 23), which reported that high GPA in science courses in high school and admission tests (MCAT) were the best predictors of later success in medical school. In the same context, Simpson et al. (24) found that the university admission index (based mainly on high school grades) had a high value in predicting overall and knowledge-based outcomes of medical school. Moreover, recent studies found that medical school admission test scores were one of the strongest predictors of academic performance later on during school years (25, 26).

Students in this study were divided according to admission type based on their performance in admission exams (English and Science tests). Students who were ineligible for enrollment in Year 1 were directed to join a foundation program to prepare them to join Year 1 of the medical school. In contrast to the students who were enrolled directly in Year 1, the HSGPA of the students who were enrolled in the foundation program showed no correlation with their performance in medical school. This might mean that admission should not depend on the HSGPA of those students and also explains the necessity of the foundation program in the rectification of the linguistic and intellectual readiness of those students to join medical school.

Regarding the performance of foundation year students, the results indicated that there is no statistically significant relationship between the HSGPA and the academic performance of all levels of study (Year 1, Year 4, B.Sc., and MD). In contrast, the results indicated that there is a positive and statistically significant correlation between the foundation year student scores in each of the Science and English language admission tests and their academic performance for all levels of study. The results indicated that the values of the correlation coefficients came to a medium degree, and this can be justified by the fact that the foundation year students study intensive courses in mathematics and the English language, and this contributed to increasing their performance in the different academic years.

Comparing the predicting value of Science test scores and English test scores revealed that the first was more predictive of Students’ performance in medical school than the latter. Moreover, the Science test was found to be more important in explaining the variation in Students’ academic performance in medical school than the English test. This was found to be true for all academic performance indicators, which are Year 1 GPA, Year 4 GPA, average M.Sc. test scores, and average MD test scores. This can be explained by the fact that the content of such Science test is very similar to the basic medical sciences studied during the first 2 or 3 years of medical school. These findings are consistent with the study of Dunleavy et al. (27), who reported that the biological and physical sciences were the strongest predictors of academic performance in medical school through graduation. They are also consistent with a study by Wilkinson et al. (28), who reported a significant correlation between six admission measures (grouped together as a biomedical science domain consisting of a human biology course, biochemistry course, cellular biology course, chemistry course, physics course, and epidemiology and public health course) and performance in medical school.

The study results indicate that both the Science test and HSGPA could predict Students’ performance in Year 1. However, it should be noted that the Science test is more predictive compared to other predictors. Also, both the Science test and English test could predict Students’ performance at the different stages (Year 4, B.Sc., and MD).

## Conclusion

The Science test results were found to be more predictive of academic performance compared to other predictors. Not all the admission criteria used for student selection are good predictors of Students’ academic performance in the medical program. The current admission criteria provide some insight into the predicted future academic performance of students. However, we believe that there is a pressing need to develop more sensitive admission criteria that can better predict student performance in both the pre-clinical and clinical years. Care should be taken to develop comprehensive admissions criteria, covering both cognitive and non-cognitive factors, to identify the best applicants to become good, future-ready doctors. Moreover, the inclusion of other valid and reliable admission tools, such as the multiple mini-interviews and the questionnaire for candidates’ suitability to follow a problem-based learning curriculum, is recommended. It would also be useful to conduct further studies to determine the predictive validity of new admission criteria. As a further research point, we recommend comparing the academic achievement of students with no foundation year with their peers who have foundation year to explore the value of such foundation year in predicting student performance in medical programs.

## Data availability statement

The raw data supporting the conclusions of this article will be made available by the authors, without undue reservation.

## Author contributions

AA and MS conceived and designed the study. AI and MS performed the research process and collected the data. AA performed the statistical analyses and was the project manager. AA, AJ, and MS wrote the original draft of the manuscript. AA, AJ, and HA prepared the figure and tables. HA, AJ, AA, and MS edited and revised the manuscript. All authors read and approved the final version of the manuscript.

## Abbreviations

AGU: Arabian Gulf University
CMMS: College of Medicine and Medical Sciences
GPA: Grade Point Average
HSGPA: High School Grade Point Average
MD: Medical Doctor
B.Sc.: Bachelor of Medical Science
MCAT: Medical College Admission Test
MCQs: multiple-choice questions.
